# Non-anesthetic Effects of Anesthetics and Organ Protection

**DOI:** 10.2174/011570159X359884250714172718

**Published:** 2026-01-08

**Authors:** Lucas Gabrovic, Xiaofeng Chen, Han Huang, Hailin Zhao

**Affiliations:** 1 Department of Surgery and Cancer, Division of Anaesthetics, Pain Medicine and Intensive Care, Faculty of Medicine, Imperial College London, Chelsea & Westminster Hospital, London, UK;; 2 Department of Anaesthesiology and Key Laboratory of Birth Defects and Related Diseases of Women and Children (Sichuan University), Ministry of Education, Children's Medicine Key Laboratory of Sichuan Province, West China Second University Hospital, Sichuan University, Chengdu, China

**Keywords:** Organ injury, organ protection, anesthesia, pharmacology, cell death, inflammation

## Abstract

Ischaemia reperfusion (I/R) injury is a physiological phenomenon whereby hypoxic tissue damage can be perpetuated by tissue reperfusion; this can occur in the setting of pathology or as a surgical complication. Naturally, tissues sensitive to hypoxic episodes such as the brain, heart, kidney, liver and lung tissue are most often affected. Current treatments for I/R injury focus of limiting the pathological response to reperfusion through ischemic preconditioning (IPC) and medications that mimic the IPC response. Anesthetic preconditioning (APC) and anesthetic postconditioning (APoC) can produce protective responses similar to IPC, thus modulating the effects of I/R injury, with a far longer impact on organ systems than their sedative or analgesic effects. The pathological process and molecular mechanism of I/R injury involve calcium overload, mitochondrial dysfunction, oxidative stress, inflammation, autophagy, and other key signaling pathways. However, how anesthetics are involved remains to be further investigated. Elucidating its underlying mechanism is vital to prevent perioperative I/R injury and benefit our patients. Importantly, the protective mechanisms differ between the types of anesthetics and between types of tissue. Understanding the differences can lead to more informed clinical decisions. Here, we systematically review and compare the molecular mechanisms that can explain how inhalational and intravenous anesthesia regulate I/R injury and provide a comprehensive analysis of recent basic clinical studies for APC and APoC in the context of different organ I/R injury.

## 1 INTRODUCTION

Acute ischaemic injury can occur in the setting of pathology, such as cerebral vascular infarcts or acute coronary syndrome, and as surgical complications such as during transplantation [1]. The only way to treat ischaemia is to restore blood flow, however, reperfusion itself can trigger further cellular damage in a process known as ischaemia reperfusion (I/R) injury. The mechanism of ischemic-reperfusion injury is summarized in Fig. (**1**). There is a growing body of evidence demonstrating that anaesthetic agents can modulate the effects of the initial ischaemic insult as well as the subsequent tissue injury caused by reperfusion. For many years the effects of anaesthetics were to be limited to sedation, analgesia, and paralysis. However, in recent decades, it has become evident that anaesthetics can have both positive and negative effects on organ systems long after their sedative effects have worn off. Anaesthetics can be broadly divided into two groups: volatile anaesthetics such as sevoflurane, isoflurane, and Xenon and intravenous anaesthetics such as propofol, benzodiazepines, dexmedetomidine, and opioids. Many of these anaesthetics have non-anaesthetic properties such as modulating cancer recurrence, neurocognitive dysfunction, and acute hypoxic tissue injury [2-4]. Anaesthetic agents have been shown to be protective against tissue damage when given before or after the I/R insult, a phenomenon called ‘anaesthetic preconditioning’ (APC) when given before the insult, and ‘anaesthetic postconditioning’ (APoC) when given after. This review will focus on summarizing our current understanding of the mechanisms involved in anaesthetic pre- and post-conditioning and the results of pertinent studies published within the last 5 years.

## 2 SEARCH METHODS

Two searches were conducted to gather data for this review. Firstly, a search for review articles related to APC for I/R protection published after January 1, 2015, was conducted to determine the current accepted fundamental mechanisms of APC. A second more robust search was conducted to identify recent preclinical and clinical studies related to anaesthetic preconditioning of neuronal, cardiac, renal, hepatic, and pulmonary tissue. An electronic literature search was conducted on the medical databases PubMed, Google Scholar, and Cochrane Library. One researcher (LG) conducted the search using key terms and Boolean operatives to identify relevant articles. The following terms were searched for in the title or abstract of articles: “anaesthetic preconditioning”, “anaesthetic postconditioning”, “anaesthetic organ protection”, “ischaemia-reperfusion”, “hypoxia-reperfusion” and “neuro-/cardio-/reno-/hepato-/pulmonary protection”. The references of potentially relevant articles were also included if deemed appropriate based on their contents. Articles were then screened based on their title and abstract and their data extracted by the researcher (LG).

The following criteria were used for the selection process: the study investigated the impact of APC following an ischaemic insult; if a preclinical study, there was a control group which didn’t receive APC; the research type was either a mechanistic study or a randomized control trial/cohort study.

Articles that met the following conditions were excluded from the review: preclinical studies where the primary outcome wasn’t tissue death, studies without a significant hypoxic insult to the tissue, studies investigating ischaemic preconditioning, clinical studies investigating specific patient subgroups and duplicate publications.

The researchers then extracted the following data from eligible articles: (1) First author, year of publication, and country; (2) Target organ tissue/cells investigated; (3) Type of anaesthetic used for preconditioning; (4) Model of I/R injury; and (5) Outcome indicators including, infarct size, biomarkers of organ function, levels of protein/mRNA expression and histopathological analysis.

## 3 THE CONCEPT OF CONDITIONING

Conditioning is a phenomenon that was first described by Murry *et al*. in the 1980s following his observation that episodes of sublethal myocardial ischaemia followed by reperfusion before a prolonged ischaemic insult resulted in a substantial decrease in myocardial infarct size [5]. The protection to prolonged ischaemia conferred by the initial exposure to sublethal ischaemia became known as ischaemic preconditioning (IPC). It was later discovered that a similar protective response can be elicited by conditioning with an anaesthetic stimulus rather than short ischaemic insults [6].

To understand how anaesthetics can protect tissues from I/R injury it is important to understand the many physiological factors causing tissue injury in the first place. Initial ischaemia disrupts cellular homeostasis by increasing the concentration of intracellular calcium which interferes with cell signalling and activates proteases that degrade cellular material [7]. Reperfusion, while necessary to prevent further ischaemic damage, further drives intracellular calcium accumulation and the opening of the mitochondrial membrane permeability pore (mMTP) which uncouples ATP synthesis and eventually causes mitochondrial swelling [7]. Reperfusion also produces reactive oxygen species (ROS) which potentiate mitochondrial dysfunction, non-specifically damaged intracellular structures (*e.g*. DNA, RNA, lipids, proteins), and interfere with cellular signaling. Inflammation is another driver of I/R injury. Initial cell death caused by ischaemia leads to the production of pro-inflammatory cytokines (*e.g*. TNF-α, IL-1, IL-6 and IL-8) from stressed cells which activate local leukocytes. Upon reperfusion these proinflammatory cytokines and activated leukocytes are swept into circulation leading to a systemic response. Local and systemic factors then promote the upregulation of adhesion molecules, vasoconstrictors, and activation of the complement system leading to a highly inflammatory response primarily in local ischaemic tissue [8]. Finally, inflammation, calcium overload, ROS, and mitochondrial dysfunction can all then modulate cell death pathways. The Bcl-2 family of proteins, which are major regulator of the apoptotic pathway, are commonly mentioned as important components of I/R injury pathophysiology [9]. Additionally, ischaemia and subsequent lack of cellular energy leads to the upregulation of autophagy which breaks down cellular components such as amino acids to facilitate energy production. While initially protective, prolonged ischaemia or further stress from reperfusion can lead to uncontrolled autophagy and autophagic cell death [9].

Anaesthetics can modulate the complicated process of I/R injury in a biphasic manner. An initial protective phase occurs shortly after the preconditioning (PC) stimulus and lasts three hours; this response is mainly driven by the upregulation of existing pro-survival proteins [10]. A second protective phase occurs 24 hours after the PC trigger and lasts up to 72 hrs; this phase is driven by the synthesis of novel cytoprotective proteins [11, 12]. The exact mechanisms through which APC protects cells from ischaemia are not clear. However, there are two main molecule pathways proposed through which APC may protect against cellular metabolic dysfunction, the reperfusion injury salvage kinase (RISK) pathway and the survival activating factor enhancement (SAFE) pathway [10]. The RISK pathway is activated by G-protein-coupled receptors and involves secondary messengers such as PI3K/Akt, nitric oxide synthase (NOS) and protein kinase C (PKC). The SAFE pathway is triggered by tumor necrosis factor-α (TNF-α) which upon binding to its receptor can activate Jak and/or signal transducer and activator of transcription 3 (STAT3) which act as transcription factors and modulate mitochondrial respiration. There is likely significant crosstalk between these two pathways and while both pathways converge to modulate mitochondrial function, they likely have many end effector responses including inhibiting of apoptosis, inducing nitric oxide synthases, and increasing antioxidant activity [13]. Ultimately, these two pathways likely do not explain the APC in full but provide a backbone which can be used to propel further study.

## 4 MOLECULAR MECHANISMS OF ANAESTHETICS MEDIATED ORGAN PROTECTION

As mentioned above, the end effectors and triggers of the APC response are poorly understood, however mechanism studies have found several potentially crucial pathways which when inhibited can diminish the effects of APC protection. The following sections will discuss our current understanding of how anaesthetic pre- and post-conditioning modulate HIF-1𝛼 signaling, cell death and inflammation to confer ischemic protection.

### 4.1 Activation of HIF-1 System

The hypoxia-inducible factor (HIF) system consists of a group of three α isoforms (HIF-1𝛼, HIF-2𝛼, and HIF-3𝛼) [14]. The stabilization of the HIF-1𝛼 isoform is involved in the primary cellular response to hypoxia which has numerous downstream effects including, angiogenesis, chromosomal stabilization, and the promotion of anaerobic respiration [14, 15]. Under normoxic conditions HIF-1𝛼 is hydroxylated by prolyl-4-hydroxylases (PHDs) which leads to its ubiquitination and degradation, a process that requires oxygen as a substrate [16]. In hypoxic conditions, PHDs cannot tag HIF-1𝛼 for degradation allowing their intracellular concentration to build. In large concentrations HIF-1𝛼 translocate to the nucleus and dimerize with HIF-1β. Formation of the HIF-1𝛼: HIF-1β complex permits binding to DNA transcriptional site targets known as hypoxia-response elements (HRE) which regulates the expression of various proteins [17]. The activity of HIF-1𝛼 can be increased by both greater protein stabilization, as in hypoxic conditions, and increased protein synthesis which can be regulated by cytokines such as prostaglandin E_2_ and insulin-like growth factor 1 [16].

Sheldon *et al*. demonstrated that hypoxic PC of the neonatal mice brain resulted in decreased histopathological damage in response to prolonged ischaemia; however, in HIF-1𝛼 knock-out mice the benefits of PC on hypoxic injury were abolished [18]. Similar results were reported by research groups investigating IPC in brain tissue, renal tubular cells, cardiac cells, and hepatocytes; suggesting that HIF-1𝛼 may be essential to the cytoprotective effects of IPC [17, 19-22]. In tissues exposed to APC HIF-1𝛼 knock-out had similar effects, however, not always [23]. Halothane, sevoflurane and propofol have been shown to decrease HIF-1α activity and the concentration of its downstream transcriptional targets in cancer cell lines, suggesting that the effect of APC on the HIF-1α system may be context specific [24, 25].

It is unclear how anaesthetics trigger the modulation of HIF-1α activity, perhaps similarly to how mammalians cells can sense the oxygen level. They can also sense the level of inhalational anaesthetic gas which may alter the cellular metabolism [26]. It has been proposed that volatile anaesthetics, specifically isoflurane, xenon and desflurane, affect the phosphorylation of proteins involved in HIF-1α degradation independent of PHDs through the inhibition of mitogen activating protein kinase (MAPK), rapamycin signaling and/or phosphorylation of PI3K [25, 27-29]. Volatile anaesthetics may also stimulate the production of mitochondrial ROS which has also been shown to increase HIF-1𝛼 activity [30]. For intravenous anaesthetics the mechanism differs. Fentanyl and remifentanil have been shown to increase HIF-1𝛼 activity though the mu-opioid receptor, a G-protein coupled receptor, a potential trigger for the RISK pathway. Even though the mechanism of HIF-1𝛼 activation is still being unveiled HIF-1𝛼 is likely essential for the anaesthetic-induced PC response of most intravenous anaesthetics as well as some volatile anaesthetics.

### 4.2 Suppression of Cell Death

Cellular death is inevitable in the setting of prolonged ischaemia where oxygen and nutrient recourses are scarce. While the initial ischaemic insult can lead to tissue necrosis, subsequent cellular death pathways triggered by reperfusion can modulate the end size of the infarct [31]. Cellular death can take different forms but can be grouped into three broad variants [32]. Type 1 cell death or apoptosis is a form of regulated cell death characterized by DNA fragmentation, cellular shrinkage, and membrane blebbing creating apoptotic bodies that can be phagocytosed by neighboring cells [32]. Type 2 cell death or autophagic cell death involves autophagy which is a process of degrading and recycling intracellular material. Hence, autophagic cell death manifests with the accumulation of autophagic vacuoles within the cytoplasm without distinct morphological changes to the cell [32, 33]. Finally, type 3 cell death or necrosis has no phagocytic or lysosomal involvement and is associated with accidental cell death triggers such as physical, chemical or mechanical stress [32].

Part of the APC response is the modulation of various pathways involved in triggering and preventing cell death. Autophagy can function as a cytoprotective mechanism as mentioned previously; however, it must be tightly regulated since autophagy of essential intracellular components can lead to autophagic cell death or apoptosis [34]. Thus, both increases and decreases in autophagy must be correlated with other experimental findings to determine their effect. The main regulator of autophagy is mTOR which functions to inhibit autophagy under conditions of growth and excess resources. Under low oxygen or resource scarce conditions mTOR activity is inhibited through REDD1 and AMP-activated protein kinase (AMPK) pathways respectively [35]. The Beclin-1 (BECN1) protein is a promoter of autophagy by controlling phagophore formation, however, in the presence of Bcl-2, BECN1 dimerizes with it inhibiting its function [35].

PC with sevoflurane was associated with increased autophagy, determined by microtubule-associated protein light chain I and II and AMPK expression, and decreased infarct size in animal hearts exposed to IR injury [36, 37]. Multiple mechanisms have been proposed explaining how anaesthetics modulate autophagic flux to promote cell survival. Shiomi *et al*. proposed that sevoflurane may cause the generation of ROS, which activates the AMPK pathway to increase autophagy [36]. Others investigating isoflurane propose that sphingosine kinase 2 is essential to increase autophagic flux in cells [38]. Xie *et al*. investigating the late PC protection conferred by sevoflurane demonstrated decreased levels of BECN1 which correlated with a reduction in autophagic cell death and increased cell survival following hypoxic stress [39]. Like sevoflurane, propofol PC conferred cardio protection associated with inhibition of autophagic cell death through the phosphorylation and inhibition of the mTOR pathway and decreased BECN1 expression [40, 41]. The ways anaesthetics affect cell autophagy have not been fully elicited, and multiple pathways have been proposed. Nonetheless, research has demonstrated that autophagy inhibitors preceding APC abolishes ischaemic protection suggesting that autophagy is likely a crucial step in the PC response [34].

Apoptosis or type 1 cell death is associated with the majority of I/R related cellular death both in the ischaemic and reperfusion phase. The regulation of apoptosis is complex but perhaps the most important regular is Bcl-2 which is why it has been a large focus of study. Bcl-2 is an anti-apoptotic protein which inhibits the activation of caspases and stabilizes the outer mitochondrial membrane preventing the release of pro-apoptotic factors. Sevoflurane PC in rats exposed to I/R injury reduced cardiac infarct size and decreased the levels of apoptosis; with associated increases in PI3K/Akt signaling and Bcl-2 family proteins [42]. The importance of Bcl-2 modulation for APC was consistently demonstrated in studies investigating PC with other volatile anaesthetics among tissue types [43-45]. Importantly, several studies have shown that at the extremes of ages and with prolonged volatile anaesthetic exposure the effects may be associated with increased apoptosis and therefore impaired neurocognitive development and longevity [46-49] An overview of the data suggests that in the setting of ischaemia volatile anaesthetics confer a neuroprotective response, however under normal or other pathological conditions the effects may be neurotoxic [48].

Intravenous anaesthetics seem to share a similar pathway of Bcl-2 modulation as volatile anaesthetics. Propofol, being one of the most used intravenous anaesthetics, has been studied in multiple tissue types. In a rat model propofol PC resulted in a lower rate of apoptosis of neuronal cells following an ischaemic insult; like volatile anaesthetic, this was associated increased concentrations of Bcl-2 [50, 51]. Similar results were seen when investigating the myocardial tissue of rats [52]. Tao *et al*. demonstrated that propofol pretreatment was associated with reduced translocation of apoptosis-inducing factor and suggest it as a further mechanism through which propofol may inhibit apoptosis [53]. Further studies link the antiapoptotic effects of propofol with its antioxidant properties and constrained opening of the mitochondrial permeability transition pore [53-55]. Opioid anaesthetics such as morphine and remifentanil were also associated with tissue ischaemic protection and decreased cellular apoptosis [56]. Mechanism studies suggest that morphine and other opioids can trigger RISK pathway intermediates to increase Bcl-2 concentration [57-59]. Studies investigating other intravenous anaesthetics such as dexmedetomidine yielded similar results albeit through stimulation of the α2-adrenergic receptor [60, 61]. Furthermore, dexmedetomidine may stimulate the PI3K/Akt pathway through the inhibition of signaling RNA fragments [62]. Peng *et al*. showed that dexmedetomidine stimulation of the α2-adrenergic receptor could affect the activity of HIF-1𝛼 which can also trigger apoptosis [63]. In summary, the majority of studies have shown that APC induced Bcl-2 upregulation is essential to prevent apoptotic cell death following I/R injury, but other pathways have also been proposed.

### 4.3 Inhibition of Inflammation

A significant element of ischaemic-reperfusion injury is the subsequent inflammation following reperfusion and cell death [64]. In general, anaesthetics are known to have an immunosuppressive effect by modulating nerve conduction involved in the pain response and the hypothalamic-pituitary-adrenal axis affecting the release of catecholamines [65]. APC has been shown to attenuate the local and systemic inflammatory response to I/R injury through additional mechanisms as well [10]. Following infarction rapid local cell death releases cellular material including proinflammatory cytokines into the local environment which leads to the activation of local neutrophils and macrophages [66]. Activated immune cells further release proinflammatory cytokines and adhesion molecules recruiting cells to local tissue and strengthening the pro-inflammatory signal. Upon reperfusion the activated immune cells can re-enter circulation leading to a systemic immune response and leukocytes not only being recruited to ischaemic tissue but also into distant healthy tissue [67]. Uncontrolled inflammation following I/R injury leads to further cell stress and death.

Anaesthetics can modulate nearly every part of the inflammatory response explained above including the initial inflammatory trigger. Nuclear factor kappa B (NF-κB) is a transcription factor that can be both proinflammatory and anti-inflammatory [64]. APC activates NF-κB before an ischaemic insult which promotes the accumulation of prosurvival proteins such as Bcl-2 and decreased expression of pro-inflammatory and leukocyte adhesion factors [42, 68, 69]. Wang *et al*. found that following sevoflurane precondition rat hearts who underwent clamping of the left coronary artery had a reduced infarct size and reduced proinflammatory cytokine expression, including Tumor necrosis factor alpha (TNF-α) and intercellular adhesion molecule-1 (ICAM-1) [42]. Importantly, infarct size returned to ‘normal’ if a NF-κB inhibitor was administered to the rats along with sevoflurane [42]. As mentioned previously NF-κB can also promote the expression of proinflammatory cytokines through nuclear translocation. Propofol PC of microglial cells, the main resident immune cell in the brain, resulted in a reduction of NF-κB expression and reduced concentrations of proinflammatory cytokines [70, 71]. Liu *et al*. preconditioned mice with propofol and then induced hepatic I/R injury and found similar results citing a reduction in NF-κB expression. Propofol preconditioned mice also experienced significantly less liver injury through dampened inflammation [72]. NF-κB activation was seen not only after infarction but before the ischaemic insult, suggesting it may play a role in APC [42].

Anaesthetics have also been associated to impair neutrophil chemotaxis, macrophage activation, phagocytosis and the generation of ROS [66]. Perhaps unsurprisingly most studies suggest NF-κB, a major regulator of the immune system, as the common factor among the different end effects [73, 74]. On the other hand, the immune response is extremely complex and as such anaesthetics likely have a vast array of mechanisms through which they can modulate inflammation, such as α2-adrenergic receptor or opioid receptors found on leukocyte membranes, which have yet to be elucidated [75].

## 5 ANESTHETICS AND ORGAN PROTECTION: LATEST STUDY AND EVIDENCE

This review will now summarize the literature regarding organ protection and APC from the last five years. It will focus specifically on neural, cardiac, renal, hepatic and pulmonary protection due to the sensitivity of these tissues to hypoxic conditions.

### 5.1 Neurological Anaesthetic Protection

Most of the research on volatile APC has been investigating sevoflurane, most likely due to its popularity among anaesthetists as an agent with little haemodynamic effects, relatively quick emergence from anaesthesia and little risk of airway compromise [76]. Multiple previous papers have demonstrated that sevoflurane PC can reduce the inflammatory response and the rates of neuronal apoptosis following I/R injury [48]. Nonetheless, the precise mechanisms of protection are not clear.

Recent studies have investigated how APC may modify neurotransmitter receptor activity to mitigate calcium toxicity following I/R injury. Neurotransmitter receptors tend to be ion channels which when dysregulated may promote intracellular ion transport including intracellular calcium accumulation. Li *et al*. found that glutamate receptor 1 (GRIA1) was downregulated in neuronal PC12 cells and the hippocampal cells of rats preconditioned with sevoflurane before I/R injury [77]. They propose that GRIA1 downregulation prevents the overactivation of the post-synaptic receptor calcium permeable-amino-3-hydroxy-5-methyl-4-isoxazolepropionic acid receptor (CP-AMPAR) which prevents intracellular calcium overload. In their model, induced overexpression of GRIA1 hindered the protection conferred by sevoflurane, suggesting that GRIA1 downregulation is a key step in the sevoflurane PC response. Wang *et al*. built on the work of previous research which identified fibroblast growth factor 2 (FGF2) as a neuroprotective molecule with the aim to identify whether the sevoflurane PC mechanism involves FGF2 [78, 79]. They induced a traumatic brain injury in rats and demonstrated that pretreatment with sevoflurane upregulated FGF2 expression, which was associated with decreased brain oedema, decreased apoptosis and protective autophagic flux. Sevoflurane pretreatment also improve the Modified Neurological Severity Score, measured through evaluation of motion, sensation, reflexes, abnormal behaviour, and balance, in rats exposed to neuronal traumatic brain injury. Further mechanism studies revealed that FGF2 overexpression upregulates enhancer of zeste homolog 2 (EZH2) which can bind to the promoter region and repress the expression of hairy and enhancer of split 1 (HES1). HES1 has been shown to promote neuronal apoptosis and worsen brain oedema following neuronal insult, while EZH2 expression can reverse the over-activation of autophagy seen following I/R injury [80]. In short, Wang *et al*. found that sevoflurane may prevent autophagic and apoptotic cell death by modulating the FGF2/EZH2/HES1 axis which may play a role in I/R injury; outlined in Table **1**.

Recent research into the neuroprotective effects of intravenous anaesthetics has focused on how these agents are able to induce increased resistance to the oxidative environment following I/R injury. Xiong *et al*. showed that rats pretreated with remifentanil or dexmedetomidine two hours before cardiopulmonary bypass displayed decreased inflammatory markers (IL-6 and IL-10) and markers of brain dysfunction (S100β and NSE) and a more favourable microscopic appearance of hippocampal tissue compared to rats not preconditioned [81]. Furthermore, on functional assessment, rats that underwent APC had reduced latency in escape from a Morris water maze and reduced time on the rest platforms indicating improved memory when compared to the rats that didn’t undergo APC. Following bypass rats had reduced expression of nuclear factor E2-related factor 2 (Nrf2) and heme oxygenase 1 (HO-1) compared to the sham group; NRF2 and HO-1 expression was not suppressed in preconditioned rats. Nrf2 is a transcription factor that when located in the nucleus can promote the expression of antioxidant molecules such as HO-1 and NQO-1. Under hypoxic conditions the PI3K/Akt pathway can promote Nrf2-induced HO-1 expression, a pathway that the researchers suggest is upregulated by sevoflurane which improves protection against oxidative stress. Nrf2, apart from its antioxidant properties, can both directly and indirectly inhibit ferroptosis, a type of programmed cell death dependent on iron homeostasis characterized by elevated iron levels, ROS accumulation, and accumulation of peroxidation product MDA [82]. Zhou *et al.* created a mice model with sepsis-induced brain damage and found that mice preconditioned with propofol had increased expression of Nrf2 and HO-1, decreased markers of ferroptosis, and abrogated neuronal loss and inflammatory gliosis [83]. Following, the researchers administered Nrf2 inhibitors or ferroptosis inducers prior to preconditioning the mice with propofol, this negated the neuronal protection previously conferred by APC. Thus, the Nrf2/HO-1 pathway and its suppression of ferroptosis are likely essential to propofol mediated neuronal APC.

### 5.2 Cardiac Anaesthetic Protection

The concept of PC began with Murry’s observations of canine hearts following I/R injury, as such the cardioprotective effects of APC are likely the most extensively studied. Many of the same pathways being researched in other organ systems such as the Nrf2 pathway or the role of sirtuin 1 in renoprotection have also been validated in cardiac models [84, 85]. Regardless, the basic mechanisms behind APC such as calcium flux, cell death, the HIF-1α system, or inflammation still need to be characterised.

Mirroring the research on neuroprotection, Yang *et al*. postulated that neurotransmitter receptors may be involved in APC preventing toxic intracellular calcium accumulation following cardiac I/R injury. Previous researchers found that vagal nerve stimulation, which acts on the heart *via* acetylcholine receptors, upregulates the calcium/calmodulin-dependent protein kinase beta (CaMKKβ) and AMP-activated protein kinase (AMPK) pathway which is associated with improved outcomes following myocardial ischaemia [86]. Yang *et al*. found that sevoflurane PC upregulated the CaMKKβ/AMPK pathway and improved the haemodynamic function of the rat’s heart, measured as an increase in left ventricle developed pressure and contractility, after I/R injury [87]. This effect could be reserved if APC was given alongside cholinergic receptor antagonists. CaMKKβ is involved in intracellular calcium flux, following, sevoflurane may modulate calcium influx following myocardial I/R injury by stimulating the cholinergic receptor. On the other hand, Zhang *et al*. suggests that sevoflurane may prevent toxic calcium accumulation by protecting the myocardial endoplasmic reticulum from stress through improving AdipoR1-Cav3 interactions [88]. The endoplasmic reticulum is a large intracellular store of calcium which when placed under stress can release calcium into the cytosol. Building on their previous research showing that caveolin-3 (Cav3), a downstream molecule of adiponectin, is key in sevoflurane mediated cardiac PC they compared wildtype (WT) and adiponectin-knock out (KO) mice preconditioned with sevoflurane [89]. They demonstrated that KO mice did not experience the benefits of sevoflurane conditioning. Additionally, WT mice with APC experienced less endoplasmic reticulum stress and increased Adiponectin R1-Cav3 interaction compared to WT mice not preconditioned; this was not seen in KO mice. Zhang *et al*. postulated that sevoflurane precondition is adiponectin dependent, and by enhancing this interaction it could decrease endoplasmic reticulum stress to mitigate intracellular hypercalcaemia.

Cell death can take multiple forms, Sheng *et al*. studied how sevoflurane PC may modulate iron homeostasis to prevent ferroptosis in a cardiomyocyte cell line isolated from rat hearts [90]. Elevated iron levels in cardiomyocytes can lead to lipid peroxidation and excessive mitochondrial ROS production. The excessive iron accumulation is postulated to lead to ROS accumulation and lipid peroxidation triggering a novel form of cell death known as ferroptosis [91]. Sevoflurane PC was found to be associated with decreased expression of proteins DMT1 and Tfr1, involved in intracellular iron transport, and increased expression of the extracellular iron transporter FPN1. A decreased in intracellular iron and MDA, a marker of peroxidation, was also observed suggesting that sevoflurane precondition may inhibit ferroptosis and regulate iron homeostasis in addition to inhibiting the classical caspsase-mediated apoptotic pathway shown by previous research [92]. Wang *et al*. investigated another type of cell death known as pyroptosis, the results of which are summarized in Table **1**. Pyroptosis is a type of cell death mediated by gasdermin D (GSDMD) or NLRP3 which involves the formation of pores in the plasma membrane releasing intracellular inflammatory cytokines into the interstitial space [93]. Wang and their team demonstrated that dexmedetomidine PC could significantly decrease infarct size and improve left ventricle developed pressure in the *ex vivo* rat heart model compared to the hearts that didn’t undergo preconditioning; no difference was observed in heart contractility between the groups. These changes were associated with decreased expression of pyroptosis-related proteins GSDMD, NLRP3, and cleaved caspase-1 in cardiac tissue. I/R injury normally upregulates the expression of miR-665 and downregulates MER2D and nuclear Nrf2 which can lead to pyroptosis. Administering a miR-665 mimic was able to abolish dexmedetomidines effects on MEF2D expression, Nrf2 nuclear translocation, and expression of pyroptosis proteins; administering a MEF2D mimic reversed the effect of administering a miR-665 mimic. Further experiments confirmed the interaction between miR-665 and MEF2D, and MEF2D and Nrf2. It was therefore proposed that dexmedetomidine can confer cardioprotection by modulating the miR-665/MEF2D/Nrf2 axis to decrease pyroptosis.

Dexmedetomidine like many anaesthetics has anti-inflammatory properties, but in the setting of cardiac I/R injury it was demonstrated to be able to specifically decrease mast cell degranulation. Xiong *et al*. excised rat hearts which were quickly perfused in an artificial incubator and then exposed to I/R injury [94]. The rats were pretreated with dexmedetomidine, mast cell degranulator C48/80, both, or nothing. Dexmedetomidine PC improved the haemodynamic parameters and arrhythmia scores and decreased mast cell activation and inflammatory cytokine release observed following I/R. Administering C48/80 had similar effects on the rats as I/R injury which were partially reversed by dexmedetomidine PC. Moreover, administering a C48/80 inhibitor and dexmedetomidine combination to rat hearts exposed to I/R injury negated the protective effects of APC, suggesting that dexmedetomidine can suppress mast cell degranulation to improve I/R injury.

A limited number of clinical trials have been conducted on cardiac APC in the last five years as previous meta-analysis have shown that volatile anaesthetics, especially sevoflurane and desflurane, are superior to intravenous anaesthetics in reducing mortality following cardiac surgery [95]. Nonetheless, further studies continue to confirm this finding. Dharmalingam *et al*. randomized patients undergoing coronary artery bypass grafting on cardiopulmonary bypass to receive either sevoflurane or isoflurane and measured cardiac function and biomarkers of oxidation during and after the procedure, results summarized in Table **2** [96]. They found that the sevoflurane group experienced more positive trends in haemodynamic parameters following surgery compared to isoflurane. Sevoflurane was also associated with an increase in the serum concentration of thiols, substrates that are quickly oxidized, both in the ischaemic and reperfusion phase of cardiac bypass highlighting sevoflurane’s antioxidant properties. Both anaesthetics prevented increases in MDA following reperfusion suggesting both anaesthetics prevented overwhelming oxidative stress, however, sevoflurane might be superior to isoflurane for cardioprotection which supports previous research.

### 5.3 Renal Anaesthetic Protection

As with other organ systems, understanding the mechanisms leading to intracellular calcium overload following renal ischaemia could help shine light on how volatile anaesthetics protect the kidney against I/R injury. Xu *et al*. investigated the divalent anion transporter transient receptor potential channel M7 (TRPM7) which is reportedly involved in cellular calcium overload following cardiac and neuronal I/R injury [97]. The researchers induced renal I/R injury *via* renal artery cross-clamping in mice, some mice were pretreated with 30 minutes of sevoflurane. Mice pretreated with sevoflurane expressed much lower levels of TRPM7 on renal epithelial cells which was associated with improved renal histology and decreased levels of blood urea nitrogen (BUN) and creatinine indicating better renal function following I/R injury. This supports the results observed in cardiac and neuronal tissue [98, 99]. Apart from TRPM7 potentially playing a role in calcium overload it can also act as an inflammatory trigger [100]. When evaluating IL-6 and TNF-α levels following sevoflurane PC, Xu *et al*. found them to be significantly reduced in comparison to mice not preconditioned. Thus, they propose TRPM7 inhibition as an important mechanism of sevoflurane renal PC by reducing calcium influx and immune activation. In addition to looking for proteins involved in molecular pathways triggered by APC, recent research has also focused on how APC may affect micro RNAs (miRNA/miR) that can modulate and trigger APC molecular pathways. MicroRNAs are short ribonucleic segments that can act directly on DNA to repress or promote expression or on proteins to affect their function [101]. Yamamoto *et al*. measured miRNA expression in the renal cells of rats that underwent renal artery clamping and found that APC and IPC modulate the expression of different miRNAs [102]. Sevoflurane PC upregulated miR-17-3p and downregulated miR27a; IPC modulated six other miRNAs. Yamamoto and his team used software to predict the effect of the miRNAs and found that the two miRNAs affected by sevoflurane PC act on the PTEN/PI3K/Akt pathway. The software predicted that miR-17 directly blocks PTEN which increased p-Akt while miR-27a directly blocks PI3K which would decrease p-Akt. The researchers confirmed these predictions using western blot analysis and concluded that sevoflurane may be renoprotective by regulating the PTEN/ PI3K/Akt pathway. Nonetheless they stated that by modulating the PTEN and PI3K molecules other pro-survival pathways may be affected such as the PI3K/caspase-3 pathway which couldn’t be ruled out in their study.

The antioxidant protein Nrf2, reported to be important in the neuroprotective effects of sevoflurane, was investigated by Wang *et al*. in a mouse and *in vitro* model of renal I/R injury [103]. Interestingly, sevoflurane preconditioning led to a decrease in serum concentrations of creatinine, BUN, and Neutrophil Gelatinase Associated Lipocalin (NGAL) demonstrating it could improve renal function in a dose-dependent manner. Improved renal function and sevoflurane preconditioning were associated with Nrf2/HO-1 pathway upregulation. The researchers then considered the immune modulatory effect of HO-1 and measured the expression of ICAM-1 and VCAM-1, vascular adhesion molecules promoting immune cell extravasation. Both adhesion molecules were downregulated in preconditioned mice suggesting that sevoflurane can decrease immune cell recruitment. Zheng *et al*. reported similar effects of desflurane on preconditioned rats exposed to renal I/R injury citing decreased cellular apoptosis, oxidative stress, and inflammatory markers [104].

There is significantly less new research on the effects of intravenous anaesthetics on renoprotection. Of note, Echevarria *et al*. investigated the effects of PC with multiple intravenous anaesthetics on sirtuin (Sirt) gene expression in a mice model with induced renal I/R injury [105]. Through histological and biochemical analysis, they demonstrated that preconditioning with morphine, fentanyl, propofol, or dexmedetomidine PC confers a renoprotective effect. Following APC all the mice demonstrated downregulated Sirt1 expression and upregulated Sirt2 expression, expect for propofol which did not modulate Sirt2 expression. They propose that Sirt2 may be essential to the preconditioning response of the aforementioned anaesthetics as Sirt2 has been shown to “attenuate NF-kB gene activation, cytokine production and apoptosis in multiple models” (Echavarría 2020), hinting at a possible mechanism for I/R injury protection.

There has been an increase in the number of clinical trials investigating APC as renoprotection is relevant not only to direct renal insults but also a major consideration during vascular and cardiac surgery. During these procedures vascular supply to the kidneys is often compromised which predisposes patients to a lengthened hospital stay and additional medical interventions. Guerrero-Orriach *et al*. demonstrated the superiority of volatile anaesthetics for nephroprotection during aortic valve replacement surgery involving the use of extracorporeal circulation (Table **2**) [106]. The researchers administered desflurane or propofol to 60 patients in total. As markers of renal function, they measured serum creatinine and urinary NGAL. Patients given desflurane had lower levels of creatinine and NGAL 24hrs and 48 hours after the procedure. A similar experimental design comparing sevoflurane to propofol in patients undergoing coronary artery revascularization found similar results [107]. This suggests that while propofol has shown renoprotective effects *in vitro* and in animal models volatile agents may be superior in the clinical setting.

### 5.4 Hepatic Anaesthetic Protection

Recent research into hepatic APC has paralleled that of other organ systems, namely studies investigating microRNAs, the Nrf2 pathway, and inflammation. The liver contains resident macrophage cells, called Kupffer cells, which likely play a role in the inflammatory reaction following I/R injury and could be involved in explaining the APC response [108]. Li *et al*. demonstrated that sevoflurane preconditioning could decrease AST, ALT and LDH concentration following I/R injury indicating improved hepatic function and decreased cell death [109]. The research suspected that this may be due to limited macrophage infiltrate into hepatic tissue following I/R injury by modulating the KLF5/ITGB2 axis. In fact, Li *et al*. found that sevoflurane preconditioned mice exhibited downregulated expression of ITGB2, decreased macrophage infiltrate, and decreased release of pro-inflammatory markers TNF-α, IL-6 and IL-1β; consistent with studies investigating cardiac tissue [110]. Mechanistically they confirmed this occurrs due to a decrease in macrophage-specific deletion of myocardin-related transcription factor A (MRTF-A) causing KLF5 and ITGB2 downregulating. Upregulation of either KLF5 or ITGB2 would negate the protection conferred by sevoflurane pretreatment on mice livers. Importantly, knocking out expression of ITGB2 in mice was able to ameliorate the effects of KLF5 upregulation suggesting that KLF5 mainly acts through ITGB2. Thus, by suppressing the MRTF-A/KLF5/ ITGB2 axis sevoflurane preconditioning may limit Kupffer cell activation and infiltration in the setting of liver I/R injury. Yang *et al*. proposed that remifentanil may modulate inflammation in a different way to sevoflurane which involves β-arrestin2 and toll-like receptor (TLR) signaling [111]. β-arrestins are negative regulators of TLRs which are involved in Kupffer cell activation but also the inflammatory pathways of MAPK and NF-κB [112]. Of the β-arrestins, β-arrestin2 binds to the opioid receptor with the highest affinity and thus proposed as a molecule involved in immunosuppressive effect of remifentanil. The researchers subjected mice to hepatic I/R injury and found TLR4 to be upregulated; preconditioning the mice with remifentanil was able to prevent the TLR4 upregulation, decrease the release of inflammatory markers and decrease the rate of hepatocyte apoptosis. Additionally, APC with remifentanil increased the plasma membrane concentration of β-arrestin2. Administering a β-arrestin inhibitor to the mice prevented the TLR4 downregulation previously seen after remifentanil preconditioning, demonstrating that remifentanil likely downregulates TLR4 *via* its interaction with β-arrestin2. Further *in silico* and *in vitro* analysis demonstrated that β-arrestin2 forms complexes with TRAF6 to inhibit the MAPK pathway which in turn downregulates the TLR4 inflammatory pathway, supporting it as a mechanism explaining how remifentanil may mitigate excessive inflammation following hepatic I/R.

Additionally, efforts have been made to understand the effect of APC on the expression of microRNAs in hepatocytes following I/R injury. Ji *et al*. researched how miRNAs may be triggers of the APC response and showed that of the six miRNAs known to be involved in hepatic I/R injury, sevoflurane can significantly downregulate the expression of miR-218-5p [113]. By downregulating miR-218 expression there was a concomitant increase in the concentration of protein GAB2, a known activator of PI3K/Akt which is involved in the RISK pathway. Induced overexpression of miR-218 attenuated the protective effects of sevoflurane, highlighting its importance in the mechanism of APC. Thus, miR-218 may be the messenger through which sevoflurane may stimulate the RISK pathway and protect hepatocytes from I/R injury. It is obvious that APC can modulate cell death pathways, how this effect is achieved is still unclear [34, 56]. A possible mechanism proposed by Xiao *et al*. stipulates that sevoflurane acts on the HGF/Met pathway to upregulate autophagy in the short-term during periods of cellular starvation and hypoxia [114]. By inducing hepatic I/R injury in preconditioned and non-preconditioned mice, they were able to correlate APC with upregulation of HGF and p-Met, the presence autophagosomes, and increased concentration of autophagy markers in interstitial fluid. Inhibiting HGF was able to negate the protective effects of sevoflurane and prevent autophagy upregulation suggesting HGF mitigates the increase in autophagy seen following sevoflurane PC (Table **1**).

There are two recent clinical studies that exist on hepatic APC which investigate whether the choice of anaesthetic during liver transplantation is associated with better graft function in the week following surgery, they are both summarized in Table **2**. A retrospective study conducted by Dieu *et al*. analysed protective factors against early graft dysfunction in pediatric patients undergoing living donor liver transplantation [115]. In the study patients received either sevoflurane, propofol or both as an anaesthetic during the procedure. The patients that received sevoflurane had a decreased likelihood of early graft dysfunction, followed by the patients that received propofol, and finally a combination of the two. Dieu *et al*. propose that the reason the combination group had the worse outcome is that APC is dose-dependent, and that this group didn’t receive a large enough dose of either anaesthetic for it to be beneficial. Nonetheless, they concluded that sevoflurane provided the best chance for improved early graft function following transplant. The second study was a randomized control trial comparing the effects of remifentanil and fentanyl during adult liver transplant surgery from deceased donors. Jowkar *et al*. found no difference in early liver dysfunction between the two anaesthetics [116]. Importantly, this study investigated APoC and not PC since the anaesthetic was administered to the graft recipient and not the donor which experienced the ischaemic phase of I/R injury.

### 5.5 Pulmonary Anaesthetic Protection

There is limited lung pathology in which I/R injury is relevant; however, it can still occur during transplantation and states of shock such as sepsis. Bertani *et al*. created a swine lung transplant model to investigate the protective effects of sevoflurane, the results of which are outlined in Table **1** [117]. Donor pigs were given either sevoflurane or no inhalational anaesthetic during the retrieval procedure, the lung was then exposed to 24 hours of cold ischaemia before being transplanted into the recipient pig. The pigs that received lungs from the sevoflurane group had stable oxygenation immediately post procedure, while the non-sevoflurane group had downwards trends in oxygenation (SpO2, PaO2/FiO2 ratio, and pH). Moreover, the sevoflurane group had more favourable histology, a much smaller rise in pro-inflammatory markers, and an increase in anti-inflammatory marker IL-10. Alessandro Bertani *et al*. concluded that sevoflurane protects the lungs from I/R injury and improved immediate post-transplant organ function.

The lungs, like the liver, are highly vascular structures, thus it could be argued that a larger consideration must be made regarding the effect I/R on the pulmonary vasculature. The endothelial barrier, which lines all vessels, can itself be injured during ischaemia but furthermore plays a role in recruiting inflammatory cells and causing lung oedema following reperfusion. Huisuo Hong and colleagues investigated how dexmedetomidine may improve pulmonary I/R injury by promoting endothelial barrier function [118]. They demonstrated that APC with dexmedetomidine conferred pulmonary protection against I/R injury in mice, which was associated with upregulated expression of keratinocyte growth factor (KGF) 2. Previous studies have demonstrated that supplementing lungs with KGF 2 improves lung oedema, inflammatory cell infiltration, and endothelial cell apoptosis following I/R injury thus Hong *et al.* suggested that this protein may be crucial in the dexmedetomidine PC response [119]. They showed that following dexmedetomidine precondition the promoter region of KGF2 is modified to increase its expression, a process normally suppressed following I/R. Through silico and *in vitro* studies they identified JMJD3 histone demethylase as the protein modifying the KGF2 promoter region during I/R injury. Induced overactivity of JMJD2 reduced the levels of KGF2 and impaired the lung protection conferred by dexmedetomidine PC suggesting it modulates the effect of remifentanil on KGF2. Summarizing, dexmedetomidine PC may protect the lungs from I/R injury by maintaining endothelial function through the JMJD3/KGF2 axis. Demonstrating yet another pathway and cell type involved in the APC response.

## CONCLUSION

There is no longer debate as to whether APC can protect the heart, brain, kidneys, lungs, or liver from I/R injury. There is however further progress to be made in understanding how APC confers I/R protection. The mechanisms of protection likely differ between volatile anaesthetics and intravenous anaesthetics, with further differences between the different types of intravenous anaesthetics. In recent years volatile anaesthetics have been shown to downregulate glutamate receptor 1 preventing toxic hypercalcemia, modulate the FGF2/EZH2/HES1 axis to promote autophagic flux, and downregulate the KLF5/ITGB2 axis to prevent macrophage infiltration, among many more. Intravenous anaesthetics have been shown to upregulate Sirt2 expression to suppress Nf-κB mediated inflammation, modify the Nrf2/HO-1 pathway to inhibit ferroptosis, and promote β-arrestin2/ TRAF6 interaction to limit TLR-triggered inflammation. This review has demonstrated the vast number of pathways proposed to be essential to the mechanism of APC. It would be highly beneficial for newly proposed mechanisms to be contextualized among the mechanisms that have already been validated to grow the shared understanding of APC rather than investigating the subject in parallel. The clinical studies included in this review show that volatile anaesthetics, in particular sevoflurane, are superior to intravenous anaesthetics in protecting early organ dysfunction following periods of ischaemia and then reperfusion. This potentially advocates for greater use of volatile anaesthetic in procedures where I/R injury is foreseeable such as transplantation, cardiopulmonary bypass and procedures where vascular cross-clamping is necessary. Nonetheless, there is need for large multi-centre randomized controlled trials to determine whether APC improves long-term outcomes following I/R injury as most clinical trials have analysed early organ dysfunction or other short-term outcomes. By promoting the integration of mechanism pathways and conducting clinical trials evaluating long-term outcomes a more holistic understanding of APC could be gained.

## Figures and Tables

**Fig. (1):**
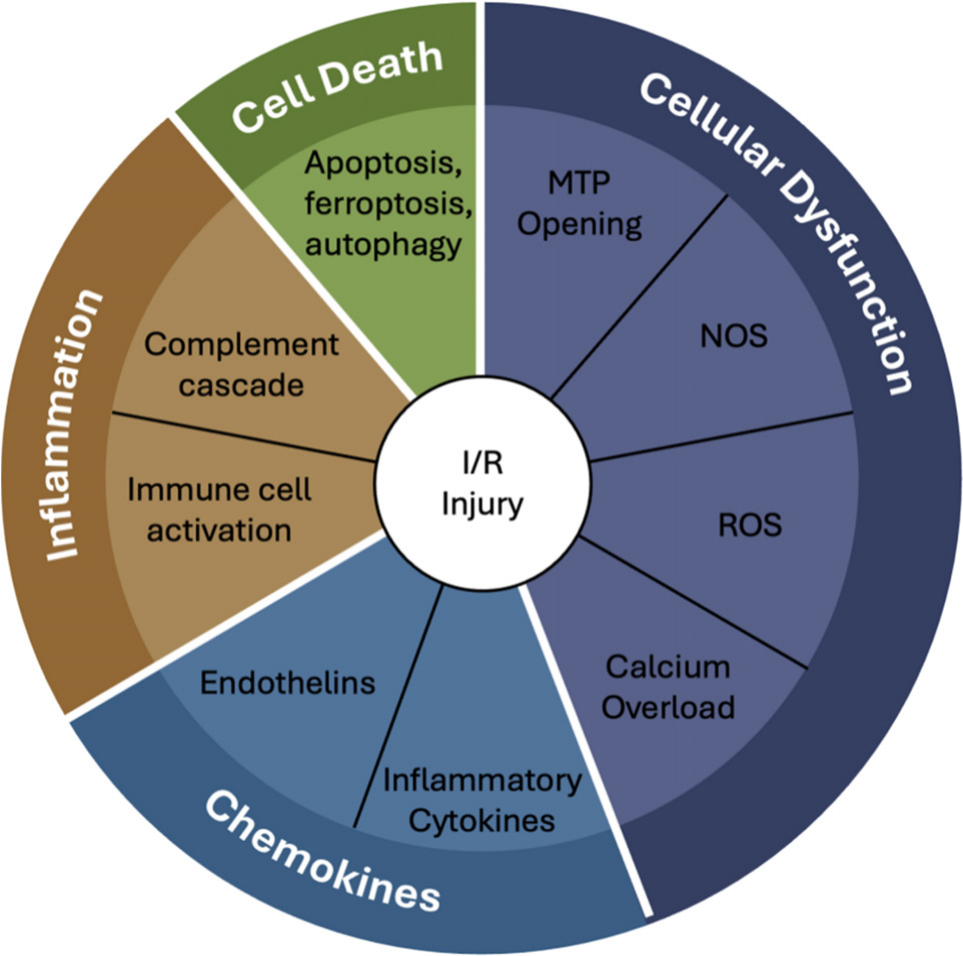
Pie diagram illustrating the different aspects involved in the pathophysiology of ischaemic-reperfusion injury. **Abbreviations**: MTP – mitochondrial transmembrane protein; NOS – nitric oxide synthase; ROS – reactive oxygen species.

**Table 1:** Summary of important publications on anaesthetic organ protection against ischaemia-reperfusion injury published in the last 5 years.

**Author (Year)**	**Study Type**	**Tissue Type** **Investigated**	**Main Findings**
Wang *et al*. (2022) [79]	*In vitro* and rat model	Neuronal	Sevoflurane preconditioning can downregulate the expression of HES1, shown to promote neuronal apoptosis and brain oedema, through the FGF2/EZH2/HES1 axis to decrease neuronal apoptosis, induced protective autophagic flux, decrease brain oedema, and improve neurofunctional status following a model of traumatic brain injury relevant to I/R injury.
Wang *et al*. (2023) [93]	Rat model and *ex vivo* rat heart model	Cardiac	Dexmedetomidine preconditioning upregulates miR-665 to downregulate the action of MEF2D and Nrf2 to decrease pyroptosis following I/R injury. This was associated with improved left ventricle developed pressure and decreased infarct size.
Xiao *et al*. (2021) [114]	Mouse model	Hepatic	Sevoflurane preconditioning is associated with HGF and p-Met upregulation which caused an increase in autophagosomes and autophagy biomarkers; as autophagy can be protected in resource scarce environments, this proved essential to the hepatoprotective effects of sevoflurane.
Bertani *et al*. (2021) [117]	Swine model	Pulmonary	The lungs of swine donors preconditioned with sevoflurane during retrieval procedures exhibited decreased markers of inflammation and more favourable histology. The preconditioned lungs functioned better with decreased central venous pressure and lactate, and increased SpO_2_ and compliance. No pigs transplanted with preconditioned lungs expired, compared to 3 in the control group. Swine donors given only thiopental and fentanyl during retrieval were used as baseline.

**Table 2:** Summary of clinical studies assessing anaesthetic organ protection from ischaemia-reperfusion injury published within the last 5 years.

**Author (Year)**	**Study type**	**Target Organ**	**Main Findings**
Dharmalingam *et al*. (2021) [96]	Randomized control trial	Heart	Sevoflurane might be superior to isoflurane for cardioprotection during bypass it was associated with a more positive trend in haemodynamic parameters following surgery. However, both sevoflurane and isoflurane prevented increases in MDA following reperfusion demonstrating the antioxidant properties of both anaesthetics.
Guerrero-Orriach *et al*. (2022) [106]	Prospective cohort study	Kidneys	Desflurane was associated with improved renal function measured through creatinine and NGAL following cardiac surgery using bypass compared to propofol suggesting the volatile agent to be superior for renoprotection.
Dieu *et al*. (2024) [115]	Retrospective cohort study	Liver	The study analysed liver function in pediatric patients that received sevoflurane, propofol, or both during living donor liver transplantation. Patients that received sevoflurane alone had a decreased likelihood of early graft dysfunction compared to the other groups.
Jowkar *et al*. (2020) [116]	Randomized control trial	Liver	Postconditioning with remifentanil or fentanyl during adult deceased donor liver transplantation had no effect on early graft dysfunction.

## LIST OF ABBREVIATIONS

APC: Anaesthetic Preconditioning
APoC: Anaesthetic Postconditioning
BUN: Blood Urea Nitrogen
HIF: Hypoxia-inducible Factor
HRE: Hypoxia-response Elements
I/R: Ischaemia Reperfusion
KGF: Keratinocyte Growth Factor
MAPK: Mitogen Activating Protein Kinase
mMTP: Mitochondrial Membrane Permeability Pore
NOS: Nitric Oxide Synthase
PC: Preconditioning
PKC: Protein Kinase C
RISK: Reperfusion Injury Salvage Kinase
ROS: Reactive Oxygen Species
SAFE: Survival Activating Factor Enhancement
TLR: Toll-like Receptor
TNF-α: Tumor Necrosis Factor-α
